# Irregularities of the Teeth and the Best Means of Correcting the Same

**Published:** 1875-10

**Authors:** Geo. W. Gray


### IRREGULARITIES OF THE TEETH AND THE BEST MEANS OF CORRECTING THE SAME.

BY GEO. W. GRAY, D. D. S.

*Read before the Oregon State Dental Society, June 30th, 1875.*

Mr. President, gentlemen and members of the Oregon State Dental Society, the subject assigned me is one of no ordinary interest; it is worthy of earnest thought and careful investigation, and is at present attracting the attention of the members of the dental profession throughout our country. Our aim should not be merely to remedy existing defects; but to act upon the principle that "an ounce of prevention is better than a pound of cure." If we can succeed in educating the public mind in reference to the means of shaping and giving symmetry and beauty to the teeth, those natural organs; for preparing the alimentary substances for the process of digestion and can secure their preservation and perfection, we will have deserved the gratitude of an appreciative public. The mission of our honored profession, is not merely to make money, but to do good in relieving pain and disease and doing all we can to secure perfection in these important organs of our physical being. We for this reason must investigate the causes of irregularity and disease that we may intelligently apply the best means of correcting the same. Allow me now to direct your attention to this subject for a very brief period while I present a few thoughts for your consideration and discussion. As to the exact period in the history of our race when these irregularities in the development of the teeth and jaws first made their appearance, causing so many hideous deformities in the expression of the human face, divine history does not furnish us any account. That they do exist and have existed for many generations past, in certain localities and among certain classes of people is an indisputable fact; and, according to the best observation I have been enabled to make during a period of fourteen years study and practice on this coast, have been unable to discover any very marked cases of irregularity, in the development or arrangement either of the jaws or teeth of the aboriginal tribes inhabiting the slopes of these western shores; and from the best evidence I have been enabled to obtain am led to conclude that these irregularities in the development of the jaws and teeth are seldom found among the aboriginal tribes of this or any other country; but exist more generally among the highly civilized, cultivated and refined classes (or upper tens) inhabiting the larger towns and cities of the world, but more especially of North America. Now the question arises why should these classes become any more liable to these irregularities than the savages or those occupying the more humble walks of life? Cannot a departure from the fixed laws of man's natural being as established by an All wise Creator be traced as the great or primary cause of the irregularities.

When God created man in physical perfection and beauty, and placed him in the garden of Eden, and told him "of every tree of the garden he might freely eat; but of the tree of knowledge of good and evil thou shalt not eat." Did he mean what he said? most assuredly. Why? Because God undoubtedly understood what was for man's good, not only in a moral point of view but physically as well, and knew if he ate of fruit of that tree he would not only be breaking his command and thus be committing a great moral wrong which would be a gross sin in itself, but it is also probable that he would be taking into his system a kind of chemical element contained in that evil fruit that would not harmonize with the natural laws governing his physical man and would thus be the means of bringing about physical disease and death, which would continue to be entailed upon the human race of all coming generations. Was man willing to obey the great law of his natural being by eating those things that Infinite Wisdom saw would result in his good for all time to come? No indeed, he saw the fruit, that it was pleasant to look upon, and good for food and that the eating thereof was calculated to make one wise, and so put forth his hand plucked and ate, regardless of consequences. And are we not continuing to follow the example set by our first parents by eating whatever is pleasing to the eyes and good for food whether in violation of nature's laws or not, and thus through a long series of generations have attained these deformities in the alveolar arch and irregularities of the teeth; a natural consequence or result of each violation. To illustrate, you will notice according to the following table by Berzelius that the bones are composed of the following chemical constituent elements.

| | | |
|---|---|---|
| Organic Matter. | Gelatin and blood vessels | 33.30 |
| | Phosphate of lime | 51.03 |
| Inorganic or | Carbonate of lime | 11.30 |
| Earthy Matter | Phosphate of magnesia | 1.16 |
| | Soda and Chloride of Sodium | 1.20 |

According to the above table, one third of man's bony frame work is composed of organic matter or 33.3 per cent. and two thirds inorganic or earthy matter or 66.7 per cent. Now in order to keep this frame work healthful and in working order you will readily perceive the necessity of of keeping up a supply of those bony elements, or mineral compounds by way of the digestive apparatus to cause a proper development of those various parts and organs; and where-ever their is a deficiency in this supply there must necessarily be a like degeneracy in the parts it was intended to supply. Are we not to conclude according to the analysis made by chemists of the present day, that the bone producing elements are very materially cut off in many articles of diet, as generally used by our fashionable livers of the present age. I will refer you to only one article, that is wheaten or fine flour bread, an article usually regarded as the staff of life, but which is more frequently made an article of death, particularly to our teeth and bony frame work. To illustrate allow me to quote from an article in the January number of Johnston's *Dental Miscellany*, of 1875 by Ephraim Cutter in which he says, that Mr. Sharples the well known chemist analyzed for him a quantity of the Peerless flour. He found 0.55 per cent. of mineral ash, a little over half of one per cent. He stated also "that the proportion of ash in the whole grain varies from 1.65 to 2.50 per cent; so that the diminution of mineral food varies from two thirds to four fifths. In other words by the use of fine flour, mankind looses from two-thirds to four-fifths of the elements that go to make up teeth and bony structure." You may argue that if this falling off in the supply of the inorganic or earthy matter would cause the alveolar arch to become small or narrow, there would also be a proportionate diminution in the size of the teeth, and thus would not become a source of irregularity. This objection at first thought seems quite plausible, but when we consider the fact that some of the worst, or most marked cases of irregularity that falls under the care of the dentist, are those in whom there is not only the narrow shriveled bar shaped alveolar arch, but also a very perceptible diminution in the size and shape of the teeth, such an argument amounts to nothing. I have noticed several cases of this nature in my own practice. One in particular now presents itself to my mind. Mr. A., about 35 years of age, enjoying ordinarily good health, of nervous temperament, seemed to have the developing power in the teeth so deficient that a number of his teeth were undeveloped altogether, while others were stunted and dwarf like in appearance, and though there was an abundance of room in the alveolar arch for them yet they came in very irregular, in almost all conceivable shapes and conditions possible, owing to these deficiencies the person was compelled to wear a partial set of artificial teeth quite early in life. And is this not likely to be the case in hundreds and thousands of instances in the not very distant future if the present dietetic system is continued! Stock raisers are very careful to study and ascertain as far as possible, the kinds of grain or food that are best calculated to develop all the good qualities of their animals, and if the bone is deficient they are sure to feed with such grain, vegetables or whatever substances contain those mineral salts more largely in order to facilitate their growth and development more fully. Now is man of so much less importance than a beast, that he should over-look all these important and valuable suggestions and be suffered to go down in premature decay and disease! Or should he not endeavor to regulate *his* diet in such a manner as will be calculated to build up and supply these deficiencies in his bony frame work, and thus lay the chief corner stone towards correcting these irregularities in the development of the jaws and teeth. In fact consider this *one* of the best, if not *the* best means of overcoming or correcting most of these irregularities in the teeth. That there are other causes of irregularities in the development of the jaws and teeth, besides this deficiency of bony material, is undoubtedly true. Such for instance as the marriage of two persons, one having very large and prominent jaws and teeth, while the jaws and teeth of the other are very small and narrow. The children of such parents frequently inherit the large teeth of the one parent and the small jaw of the other, then there would not be room in the arch for the teeth to take their places properly and they must of necessity be irregular. In such, and perhaps many other cases, mechanical means have to be resorted to, for their correction. In the application of these means there are many things to be taken into consideration. Such as the general health of the patient, nature of the alveolar processes, age, etc., etc., from 12 to 17 years being generally considered the best time to proceed with this class of operations. Many of the more simple forms of irregularity may often be corrected by the patients themselves, (by very simple means) if properly instructed. One very good way where there is any of the superior incisors, or even a canine standing a little outside the line of the arch, is by the use of a little stick cut the proper length with a notch in one end in which the tooth may rest with the weight of patient's head, the other end of stick resting on a table (or bench if at school) during hours of study or when reading or writing. By this method if carefully followed the protruding tooth will generally be driven to its place in the arch in a few days or weeks. Rubber tubing will accomplish much, also the gold and silver bars and silk ligatures will be found excellent. If it becomes necessary to enlarge or widen the arch the frequent application of rubber between the teeth will frequently accomplish the desired end. The jack screw is also very convenient for this purpose. If plates must be the final resort, the one as devised by Dr. J. B. White will prove excellent, also the one invented by Dr. A. Westcott may be used advantageously. Plates of various sizes, shapes and styles in connection with silk ligatures and and india rubber tubing or rings will be found very useful in the correction of the irregularities of the teeth, each case presented being different from every other. The opeerator will be compelled to weigh all the leading phases bearing on the subject and accept of such principles and modes of procedure as he may consider best calculated to accomplish the desired result.

